# Supplementary material for: Clustering of health behaviors among Japanese adults and their association with socio-demographics and happiness
Source: PLoS One. 2022 Apr 14;17(4):e0266009. doi: 10.1371/journal.pone.0266009 (PMC9009612; doi:10.1371/journal.pone.0266009)
Supplement: Attachment — Submitted filename: 210913_reviewer_comments.docx [file pone.0266009.s001.docx]

Point-by-point responses

**Reviewer #1**

Lines 62-64 – Confusing, please re-write

I appreciate your comment. The Introduction section has been generally revised to make its arguments and content clearer. Lines 62-64 of the original draft were revised as follows [Lines 64-67].

Despite the plethora of information on happiness and the growing number of studies across the globe finding that greater engagement in a variety of desirable health behaviors is linked to higher happiness, few studies have examined the associations between health behaviors and happiness in the Japanese population [26, 27].

-------------

Lines 65-68 this paragraph suggests that health behaviors, both + and -, may co-occur, but the evidence cited is for clusters of unhealthy behaviors. Please rewrite

Thank you for bringing this to my attention. Lines 65-68 of the original draft were revised as follows [Lines 70-74].

Indeed, some research in Europe and the United States of America have found that multiple healthy behaviors tend to occur interrelatedly and that the degree of health consciousness has a cascading effect on all types of health behaviors [28-30]. In other words, one healthy behavior can trigger a chain reaction of other healthy behaviors, and the contrary is also true.

---------------

references 3-34 are the only two that dichotomized lower vs high risk lifestyle behaviors and inspected the association with well-being, which is not setting the bar too high.

Thank you very much for this commentary. I reviewed these references and corrected their citations; specifically, the reference numbers were changed: 33 to 36, 34 to 37 [Lines 77-81].

Multiple health behaviors have synergistic effects, and therefore research on the clustering of multiple health behavior patterns and their relationship to health outcomes is increasing [28, 30, 32-36]. Further, studies have analyzed the synergistic effects of multiple health behaviors on happiness [23, 37], but findings on these effects remain scarce and they have not been examined in Japan.

--------

Lines 72-74 You need a reference backing up your statement

Thank you for this remark. Lines 72-74 in the first draft were revised as follows [Lines 59-63].

In health research, there have also been numerous studies identifying factors associated with happiness, including morbidity and mortality [4, 14], absence of disease [4], and subjective health and psychological health [15]. Various health behaviors have also been found to be associated with happiness, such as being physically active [16, 17], consuming vegetables and fruits [18-20], not smoking [21, 22], and consuming alcohol in moderation [23-25].

-------------

Line 135-138 – explain how age and SES were entered/defined. Was age entered as a continuous variable? All your other variables are dichotomized, why not age? Your SES break-down has no variability (High 94%), this is problematic, could you break down at a different point? Please explain the rationale.

Thank you for bringing this to my attention. Since I had entered age into the model as a continuous variable without sufficient consideration, I categorized and reanalyzed the age distribution. I have also reviewed the distribution of SES, the SES categories, and recreated categories based on quartiles [Lines 169-173].

Based on age, the participants were divided into three groups: younger (27-44), middle (45-55) and older (56-65). Regarding SES, participants were asked to describe the disposable income per household, so the responses were divided into three groups based on the quartile: lower (<2,150 K, where K is 1,000 yen), middle (2,150 K to<4,040 K) and higher (≥4,040 K).

------------------

After Line 188 – Table 2
Your labels are wrong from Vegetable intake to Physical activity – your % add to 100%, but your descriptors suggest otherwise (e.g should be Every day vs. 6 or less days a week)

Thank you very much for this commentary. Table 2 indicated the raw number and the percentages for participants with higher risk behavior or lower risk behavior. I have now revised the labels in the Table to ensure greater clarity for the classification of higher risk behavior and lower risk behavior by category.

---------------

Lines 207-210 Age was a significant predictor of cluster 1, but you do not mention it in the results or the discussion.

Thank you for your commentary. Although I did discuss about the relationship between age and the Clusters, my discussion about this topic was insufficient and was unclear. Thus, I partly revised Lines 207-210 in the original draft as follows [Lines 284-295].

Indeed, as people get older, they develop more health awareness and attempt to engage in healthier behaviors and lifestyles [32,61]. Among the Japanese people, those older tend to have more health awareness and adopt healthier lifestyles compared with younger people [41,62]. Still, other research revealed that people in their 30s to 50s are less likely to be aware of their own health because of increasing roles and responsibilities in both family and work life [41,63], while younger people have lower awareness since they do not have subjective symptoms of chronic or other diseases [64,65]. Moreover, studies showed that to reduce the risk of non-communicable diseases (e.g., diabetes, hypertension, or cardiovascular diseases) it is important to take measures aimed at changing multiple health behaviors [66,67]. Therefore, since health-promoting behaviors may have a significant impact on the future health status of young people, it may be necessary to develop and promote health awareness-related initiatives among the younger groups of the Japanese population that encourage their engagement in health-promoting behaviors.

---------------

Line 316 remove the word “mainly”

Thank you for your advice. I have followed your recommendation and removed ‘mainly’ from this sentence.

------------------

In limitations discuss your low response rate (valid response rate: 31.1%) and the high-income level of your sample.

I appreciate your comment and advice. I added the following description about the low response rate in my study and the aforementioned sample bias to Lines 359-363. Regarding SES, I have reconfirmed and the distribution and category division, recreating them based on quartiles. Thus, I saw no need for including more information regarding the topic of SES in the Limitations section [Lines 361-365].

Second, the valid response rate was relatively low in this study; since I used data only from a single wave (i.e., 2017) of a long-term panel dataset, it is a reality that the youngest participants at baseline are getting older. Moreover, a certain percentage of participants dropped out, and I cannot rule out the possibility of sample attrition bias. Therefore, caution should exerted when interpreting the study findings.

------------------

Line 222-227 – In your discussion do not overstate your findings, you cannot prove causality nor directionality. Expand the implications of using cross-sectional data ONLY.
Acknowledge the efforts to evaluate directionality and its implications. The few studies that have examined the directionality within the happiness-health relationship appear to support the existence of the Top-Down Effect that suggests that happiness (a predisposition to color your life experiences) may directly impact health. However, studies that have provided evidence for the Top-Down Effect (Happiness may be impacted by our life experiences) have had test populations of primarily young adults (Kong, Hu, et al., 2015; Ngamaba et al., 2017; Schwerdtfeger et al., 2017). In contrast, the few studies that have examined the relationship in older adults (over the retirement age) provide some evidence of a Bottom-Up Effect (Gana et al., 2013 and from your reference list Moriyama et al). Several researchers suggests there is support for a bidirectional model (Feist, Bodner, Jacobs, Miles, & Tan, 1995; Diener, Lucas, & Scollon, 2006; Schimmack, Schupp, & Wagner, 2008).

I appreciate your commentary and advice. I agreed with your appointments, so I have revised the Discussion and the Limitations section as follows [Lines 344-355] and [Lines 358-360].

As the current study was cross-sectional, the causal relationship between health behaviors and happiness could not be examined. However, the current study confirmed the relationship between high levels of happiness and healthy behaviors and showed that health risk behaviors were more prevalent among those who had jobs or had lower SES. If healthy behaviors lead to higher levels of well-being as indicated in previous research [23, 37], it may be important for stakeholders to endeavor to assist the structuring of the private and work lives of employed Japanese individuals in a way that enables employees to adopt these healthy behaviors, a goal for which policy interventions may be required.

Meanwhile, although engaging in health behaviors may generate positive emotions and increase happiness, it is also possible that people engage in health habits because they are happy and have a positive psychology. Therefore, further research is needed to elucidate the underlying mechanism of the relationship between health behavior and happiness.

**Limitations**

Regarding study limitations, first, the analyses were based on cross-sectional data; thus, further investigation using longitudinal data is needed to understand the causal relationships between happiness and health behaviors.

--------------------

Suggested references to add

I greatly appreciate your advice. I reviewed the References section as per your suggestion and reviewed other relevant literature. This resulted in my study now citing some of the references you have provided and some additional studies I have reviewed.

1. Feist, G. J., Bodner, T. E., Jacobs, J. F., Miles, M., & Tan, V. (1995). Integrating Top-Down and Bottom-Up Structural Models of Subjective Well-Being: A Longitudinal Investigation. Journal of Personality and Social Psychology, 68(1), 138–150. doi: 10.1037/0022-3514.68.1.138
2. Gana, K., Bailly, N., Saada, Y., Joulain, M., Trouillet, R., Hervé, C., & Alaphilippe, D. (2013). Relationship between life satisfaction and physical health in older adults: a longitudinal test of cross-lagged and simultaneous effects. Health Psychology, 32(8), 896.
3. Leonardi, F., Spazzafumo, L., & Marcellini, F. (2005). Subjective well-being: The constructionist point of view. A longitudinal study to verify the predictive power of top-down effects and bottom-up processes. Social Indicators Research, 70(1), 53–77. doi: 10.1007/s11205-005-5016-7
4. Ngamaba, K. H., Panagioti, M., & Armitage, C. J. (2017). How strongly related are health status and subjective well-being? Systematic review and meta-analysis. European Journal of Public Health, 27(5), 879–885. doi: 10.1093/eurpub/ckx081
5. DIENER, E, LUCAS, R E, & SCOLLON, Christie N. (2006). Beyond the hedonic treadmill: Revising the adaptation theory of well-being. American Psychologist, 61(4), 305-314. <https://ink.library.smu.edu.sg/soss_research/9> 21
6. Schimmack, U., Schupp, J., & Wagner, G. G. (2008). The influence of environment and personality on the affective and cognitive component of subjective well-being. Social indicators research, 89(1), 41-60.
7. Røysamb, E., Tambs, K., Reichborn-Kjennerud, T., Neale, M. C., & Harris, J. R. (2003). Happiness and health: Environmental and genetic contributions to the relationship between subjective well-being, perceived health, and somatic illness. Journal of Personality and Social Psychology, 85(6), 1136–1146. doi: 10.1037/0022-3514.85.6.1136
8. Koivumaa-Honkanen, H., Honkanen, R., Viinamäki, H., Heikkilä, K., Kaprio, J., & Koskenvuo, M. (2000). Self-reported life satisfaction and 20-year mortality in healthy finnish adults. American Journal of Epedimiology, 152(10), 983–991.
9. Koivumaa-Honkanen, H., Kaprio, J., Honkanen, R. J., Viinamäki, H., & Koskenvuo, M. (2005). The stability of life satisfaction in a 15-year follow-up of adult Finns healthy at baseline. BMC Psychiatry, 5, 1–8. doi: 10.1186/1471-244X-5-4
10. Lyubomirsky, S., King, L., & Diener, E. (2005). The benefits of frequent positive affect: Does happiness lead to success? Psychological Bulletin, 131(6), 803–855. doi: 10.1037/0033-2909.131.6.803

**Reviewer #2**

Abstract
1. It would be helpful to split the abstract into subsections (intro, method, results, discussion) to make it easier to read.

I appreciate your advice. Since I concur with you, I have added subsections to the Abstract.

2. ‘In total, 86.4% participants showed health behaviours simultaneously’ – I think this phrase needs rewording as it is not clear what is meant by it.

I appreciate your comment. After due consideration, I deemed that including a discussion on the relationship between the number of unhealthy behaviors and well-being in the current paper would make the argument of the manuscript somewhat confusing; thus, I decided not to include this topic in the study. However, I overlooked a portion of the content related to this topic that needed to be excluded; I have now removed this sentence, and I apologize for this mistake.

3. Are the confidence intervals 95% CIs? This needs stating.

Thanks for this remark. I revised “CI” to “95% CIs” throughout the whole paper.

4. You mention that participants who engaged in more than four behaviours and then report 3 Odds ratios – what do these relate to?

Thank you very much for this question. After due consideration, I deemed that including a discussion on the relationship between the number of unhealthy behaviors and well-being in the current paper would make the argument of the manuscript somewhat confusing; thus, I decided not to include this topic in the study. However, I overlooked a portion of the content related to this topic that needed to be excluded; I have now removed this sentence, and I apologize for this mistake.

5. The conclusion seems to state that the associations are causal – by increasing health behaviours, then people will be happier. But this could also be the other way around, where happier people are more likely to perform health behaviours

I would like to show my appreciation for your advice. Since I agree with you, I have revised the Conclusion of the abstract to concur with your appointments, as follows.

Conclusion: This study identified the clusters of health behavior patterns among Japanese adults, and an association between the lower health risk cluster and higher happiness was indicated. However, the causality of the relationship between health behavior and happiness was not clarified, highlighting the need for further research to elucidate its underlying mechanisms.

Introduction
6. It would be useful to define happiness as it has been used in other studies – your introduction seems to state that it is a sub factor of wider wellbeing, but it would be useful to understand how it is being operationalized in the paper.

Thank you very much for this commentary. I now added a definition of happiness, as follows [Lines 50-54].

Conceptually speaking, happiness refers to one’s overall appreciation of life as a whole and to a subjective state of mind [9], and has often been deemed as an ultimate life goal [5]. According to Diener [10], happiness is synonymous with subjective well-being and can be regarded as a global term for the various types of evaluations that people make, both positive and negative, about their lives.

7. You could go further when describing how health behaviours may be interrelated, at the moment it feels like the introduction as a whole is lacking in some depth and could go further in the justification of why the study is important and why you have focused on these specific variables to look at.

Thanks for this suggestion. I have generally modified the Introduction section based on your commentaries as well as those from Review 1. The part you pointed out was revised and added in the Introduction [Lines 68-81] and Variables section [Lines 110-114] as follows.

**Introduction:**

In addition, many studies have investigated the relationship between individual health behaviors and physical or psychological health status in isolation; nonetheless, I know that lifestyles are made up of multiple health behaviors. Indeed, some research in Europe and the United States of America have found that multiple healthy behaviors tend to occur interrelatedly and that the degree of health consciousness has a cascading effect on all types of health behaviors [28-30]. In other words, one healthy behavior can trigger a chain reaction of other healthy behaviors, and the contrary is also true. Moreover, the simultaneous occurrence of multiple health behaviors was attributed to various mechanisms, including the commonality of multiple factors associated with the behaviors, and the fact that one behavior may be a coping strategy for another [31].

Multiple health behaviors have synergistic effects, and therefore research on the clustering of multiple health behavior patterns and their relationship to health outcomes is increasing [28, 30, 32-36]. Further, studies have analyzed the synergistic effects of multiple health behaviors on happiness [23, 37], but findings on these effects remain scarce and they have not been examined in Japan.

**Variables:**

The health behaviors that have been emphasized in previous studies are smoking, alcohol consumption, diet, and exercise [28-30, 38-40], with more recent ones having focused on the relationship between vegetable and fruit intake and physical and psychological health [18-20, 28-30]. In addition, studies have analyzed whether or not people have the habit of eating breakfast and how this associates with one’s physical and psychological health [28, 29].

8. Why focus on happiness? Why not use general wellbeing or a negative psychological variable such as distress? You mention that some other studies have looked at happiness, but this seems to be a very broad factor to focus on and I think you need to go further with why you decided to focus on this.

I sincerely oblige your questionings. I have generally modified the Introduction section based on your commentaries as well as those from Review 1, modifying the initial paragraph, Lines 48-56, to be as follows. I hope it has given space for the questions you have given rise to above to be discussed.

Worldwide, the term “happiness,” which has been considered as a key element of individuals’ well-being, productivity, and quality of life, has received much attention [1-8]. Conceptually speaking, happiness refers to one’s overall appreciation of life as a whole and to a subjective state of mind [9], and has often been deemed as an ultimate life goal [5]. According to Diener [10], happiness is synonymous with subjective well-being and can be regarded as a global term for the various types of evaluations that people make, both positive and negative, about their lives. In general, happiness is a concept related to well-being that can be comprehensively evaluated regarding its positive and negative aspects; thus, it may serve as an effective indicator for measuring individuals’ overall satisfaction with their situation in life.

9. You refer to ‘human capital’ but don’t explain what this is – I am unfamiliar with this term and so it would be useful to explain what you mean by this and why it is important.

Thanks for this bringing this to my attention. I have generally modified the Introduction section based on your commentaries as well as those from Review 1; during the process, the term “human capital” was removed.

Method
10. The data used in this paper is from 2017 – is this the most recent data from these household surveys?

Thanks for this question. Yes, the 2017 dataset is the most recent data available at the time I wrote this manuscript. However, the 2019 dataset is ready for use. So, I will confirm the replication of this study in the future.

11. The questionnaire refers to ‘recent’ alcohol consumption – were participants given a more specific timeframe than this (i.e. In the past month or six months)? If not, then participants timeframes when answering this question may have differed from each other.

Thanks for highlighting this topic. The survey data did not indicate a specific timeframe for the alcohol consumption measurement; thus, I have added the following information to the Limitations section [Lines 366-371].

Third, this study was based on a secondary data analysis; namely, I could not escape the constraints imposed by the chosen study design, especially regarding the measurement tools used by the questionnaire survey. Some of the problems that they have include unclear timeframes for measuring alcohol consumption, having items about physical activity that did not comprehensively inquire about exercise amount and intensity, or about vegetables and fruit intake that did not comprehensively inquire about the specific amounts that participants ate.

12. Were participants given a comparison amount to understand the ml of alcohol or were they asked about the type of alcohol? – 180ml of a spirit is a very different amount compared to wine, or does this refer to the alcoholic content of the drink? More detail is needed here. Also, is the 23gm of alcohol a standard measure of alcohol consumption? Most of the alcohol research I am familiar with uses units rather than grams in this way.

Thank you for your comment. Respondents were asked to convert their answers regarding drinking to the amount of sake that this would represent; namely, their drinking quantities of other types of alcoholic beverages were to be converted to what they would represent based on a sake bottle. This allowed for calculating the alcohol content that they consumed. To further describe this in the text, I have added the follow description to the Variables section [Lines119-126].

Alcohol consumption was assessed using two questions. The first question was “How was your recent alcohol consumption habit?” Which had a scale with the following responses: never, a few times a month, 1−2 times a week, more than three times a week, and every day; the second question was “What is your average alcohol consumption in day when converted to sake?” Which had a scale with the following responses: less than 180 ml, 180−360 ml, 360−540 ml, 540−720 ml, 720−900 ml, and more than 900 ml. As sake contains 23 g of alcohol per 180 ml, responses were coded as 0 for “more than 23 g of alcohol daily” and 1 for “no-alcohol consumption or less than 23 g of alcohol daily.”

13. Participants were not questioned about the amount of cigarettes so you cannot differentiate between heavy vs. light smokers which is a limitation. As a whole, I think because you are using a secondary source of data, you are limited to the questions that were asked and this needs to be considered in the discussion.

Thanks for this commentary. In this survey, there seemed to be no consideration regarding the number of cigarettes smoked; however, I have referred to the WHO’s view, which describes that smoking even one cigarette per day can have a negative impact on health [Lines 127-131].

Smoking status was assessed using a single question: “Do you smoke cigarettes?” Which had a scale with the following responses: I smoke, I smoke sometimes, I have quit smoking, and never. The World Health Organization (WHO) recommends people to not smoke tobacco in order to maintain a good health [42, 43]; responses were coded as 1 for “non-smokers,” as those who were not current smokers, and 0 for “smokers,” as those who were current smokers.

14. Is the dichotomy for fruit and veg consumption none vs. any? This needs to be stated more clearly in the Variables.

Thanks for this question. I have added the following sentence to Line 136-137 to ensure greater clarity: Responses were coded as 0 for “less than 2 times daily” and 1 for “more than 2 times daily.”

15. Again, there doesn’t seem to be a question on how much exercise participants did on the days they exercised so you cannot differentiate a ten-minute workout to one lasting an hour. – this is a limitation of the questions used in the survey.

Thanks for this description. The participants were not asked to provide responses about the amount and intensity of exercise in which they engaged in; thus, I have added the following description to the Limitation section [Lines 366-371].

Third, this study was based on a secondary data analysis; namely, I could not escape the constraints imposed by the chosen study design, especially regarding the measurement tools used by the questionnaire survey. Some of the problems that they have include unclear timeframes for measuring alcohol consumption, having items about physical activity that did not comprehensively inquire about exercise amount and intensity, or about vegetables and fruit intake that did not comprehensively inquire about the specific amounts that participants ate.

16. Happiness is a single-item measure that was split using a median split – this needs to be mentioned in the limitations section. Were participants given any reference information to better understand what was meant by happiness in the study?

Thank you for your comment. The analyzed surveys did not provide further information about the term “Happiness.” Therefore, I have added the following description to the Limitation section [Lines 371-376].

Moreover, the original surveys from which my data were extracted assessed the happiness construct through a subjective, single-item scale that relied solely on self-reported responses; thus, this variable was not comprehensively explored, making it necessary for future research to use a multi-dimensional objective instrument that yields data which may allow for the development of more effective and successful strategies.

17. On line 156, p8 you mention depression but this has not been mentioned previously.

Thank you very much for highlighting this gap, and I sincerely apologize for the lack of information on the topic. I added the following description to the Variables section [Lines 155-162].

***Psychological health***

As psychological health can influence health behaviors and happiness, I used participants’ depression level as a control variable. Psychological health was measured using the Japanese version of the 12-item General Health Questionnaire (GHQ) [45, 46], and each item was rated using a 4-point Likert scale. To assess respondents’ depression level, the score categories (1= ‘less than usual’, 2= ‘no more than usual’, 3= ‘rather more than usual’, and 4= ‘much more than usual’) were converted into binary values (0, 0, 1, and 1), where higher totals score indicated more severe psychological distress. The depression for cut-off scores between 3 and 4 was used [45, 46].

Results
18. The total participant number is reduced down to 1554, presumably due to missing or invalid data – where was the data missing which meant that you could not use all participants in the analyses? Is this sample still representative once you account for the missing data? – The original household survey age range was 20-69, however in your sample they are now 27-65 so I wonder if younger people are still represented in the analyses.

I appreciate your comment. Since participants with incomplete data were excluded, I analyzed the responses of 1,554 completed questionnaires (valid response rate: 31.1%; this is described in the results section). I have now added the following description about low response rate and sample bias to the Limitation section [Lines 361-365].

Second, the valid response rate was relatively low in this study; since I used data only from a single wave (i.e., 2017) of a long-term panel dataset, it is a reality that the youngest participants at baseline are getting older. Moreover, a certain percentage of participants dropped out, and I cannot rule out the possibility of sample attrition bias. Therefore, caution should exerted when interpreting the study findings.

19. Only 6% of participants report low SES – I think this is important as we know that SES is associated with health behaviours. Does this indicate that the sample may not be representative of the whole Japanese population?

Thank you for this question. Based on peer review comments, I have reconfirmed the SES distribution, reexamined its categorization, and recreated categories based on quartiles. This highlighted a significant association between higher SES and health behaviors. Accordingly, I added information about this topic in Lines 234-238 and in Lines 312-316.

**Results:**

Meanwhile, participants who were married (OR=1.521, 95% CIs=1.130−2.047, p=0.006), had higher education (OR=2.333, 95% CIs=1.842−2.955, p=0.000), and had higher SES (OR=1.483, 95% CIs=1.083−2.030, p=0.014) had higher odds of belonging to Cluster 1.

**Discussion:**

The literature has already thoroughly discussed the socioeconomic gradient in health outcomes and health behaviors [77]. Specifically, multiple healthy behaviors were more prevalent among those with higher SES [69,78], whereas those with lower SES showed a greater prevalence of multiple health risk behaviors [28,69]. The sample of the current study confirmed these associations between multiple health behavior and SES.

20. 44% of participants report depression – this seems very high to me, what was the measure of depression used? I think it would be worth discussing whether you expected nearly half of the sample to report depression in the paper.

I appreciate your suggestion. After reviewing the GHQ cut-off points, I observed that 33% of all respondents showed depression. Still, I used GHQ only as a control variable, mostly because I assumed that the mental health status could affect happiness and health behaviors. Thus, I provided a description at the beginning of the Discussion about the characteristics of depression among the study sample, as follows [Lines 247-251].

This study showed that 33% of the participants had depressive tendencies. In a recent study, around 25% of the Japanese adults were estimated to suffer from mental health problems, including anxiety disorders and mood disorders [49]. Therefore, although the prevalence of depression in the sample of the present study was slightly higher than that for mental health problems in the Japanese population, it did not deviate significantly from the trend.

21. It would be useful to report the Ns in the text when discussing the health behaviour prevalence information.

Thanks for this remark. To address your commentary, I have added the numbers related to Cluster 1 and Cluster 2, and a Table for health behavior prevalence in both clusters: “Cluster 1 (n=817; 52.3%)” and “Cluster 2 (n=737; 47.7%).” I have also included Fig. 1 with data on the topic.

22. I think it would be useful to flip around the physical activity percentage as all the other percentages you report relate to healthy rather than unhealthy behaviours – 18% of participants reported physical activity on more than 2 days per week.

Thank you for your advice. I revised this topic as follows [Lines 210-214].

The prevalence of health behaviors is presented in Table 2. In total, 76.9% of the participants were non-smokers, and 38.5% reported no alcohol consumption or <23 g of alcohol consumption a day. Further, 46.2% ate vegetables more than 2 times daily, 7.1 % ate fruits more than 2 times daily, 75.3% reported having the habit of eating breakfast every day, and 17.8% reported engaging in physical activity more than two times a week.

23. Why are fruit and vegetable consumption reported separately and not combined into one measure?

Thank you for your pointing this out and providing this question. Vegetable intake and fruit intake were considered separate variables because they are not necessarily correlated in terms of quantity, frequency, or intake patterns, and it has been suggested that they may vary independently.

Reference: Ministry of Health, Labour and Welfare. National Health and Nutrition Survey [Internet]. [cited 2021 Sept 2]. Available from: https://www.mhlw.go.jp/content/10900000/000687163.pdf (in Japanese).

24. I’m not sure about the labelling of Cluster 1 as ‘moderately healthy’ – the label you use for cluster 2 is more descriptive whereas ‘moderately healthy’ seems to be more of a judgement. Looking at the behaviours reported, I’m not sure I would classify these individuals as ‘moderately’ healthy. In my opinion, it would be better to use a more descriptive label for cluster 1.

I appreciate your suggestion and agree with you. I have, thus reconsidered the label for Cluster 1 based on the results, having revised the topic as follows [Lines 219-222].

Cluster 1 (n=817; 52.3%) was labelled “low risk, poor fruit and exercise,” with participants in it being characterized by the lowest probability for smoking, moderately low alcohol consumption, the highest vegetable intake, regular breakfast intake, as well as low fruit intake and physical activity.

25. P11, line 197, ‘high level of inadequate alcohol consumption’ – you refer to ‘inadequate alcohol consumption’ in a few places and I think this needs to be reworded to ‘higher level of alcohol consumption’ or similar.

Thank you for this remark. I have reworded “high level of inadequate alcohol consumption” to “high level of alcohol consumption.” [Line 269]

26. What is the comparison group used for employment status?

Thank you for your comment. As per your remark, I have added the comparison group, “non-employed,” in the Table.

27. Lines 216-217, p12, need to be clear that these results relate to 4, 5 and 6 health behaviours. Currently this is unclear.

I appreciate your comment. After due consideration, I deemed that including a discussion on the relationship between the number of unhealthy behaviors and well-being in the current paper would make the argument of the manuscript somewhat confusing; thus, I decided not to include this topic in the study. However, I overlooked a portion of the content related to this topic that needed to be excluded; I have now removed this sentence (Lines 216-217, p12 in the first submitted manuscript), and I apologize for this mistake. I have also deleted the relevant table.

Discussion

28. I think it is worth pointing out that while cluster 1 refers to ‘healthier’ behaviours than cluster 2, neither group is particularly healthy and participants in the study tended to report fairly low levels of health behaviours.

I appreciate your advice. Accordingly, I have added the following description to the Discussion section [Lines 255-257].

Based on the distribution of health behavior implementation, participants in Cluster 1 showed lower health risk behaviors than in Cluster 2, although those in Cluster 1 were not completely healthy.

29. It would be worth discussing how representative the sample is of the Japanese population when you consider the participants whose data were analysed – the youngest participant was 27 and the vast majority of participants report high levels of SES, so I wonder if the results have issues with self-sampling bias.

Thank you very much for this commentary. Based on peer review comments, I have reconfirmed the SES distribution, reexamined its categorization, and recreated categories based on quartiles. This led me to conclude that there was no need add a description regarding this topic in the Limitation section.

30. You mention that stress = poorer health and that this is likely to be related to both health behaviours and happiness. Do you think that by measuring happiness, this is a kind of proxy measure where you are actually assessing stress levels?
You also mention that by increasing health behaviours, this may increase happiness. But I wonder if the issue is actually that people are too stressed to carry out health behaviours and this reduces happiness, therefore wouldn’t reducing stress be the key factor to target in order to have the biggest impact over health and wellbeing?

I appreciate your comment and advice, and I strongly agree with you. I do not think that happiness is a proxy measure of stress level. Moreover, since my study could not clarify the causality between health behaviors and happiness, the description I provided was somewhat misleading. Thus, I have revised the Discussion section to concur with the discussions you have brought forth, as follows [Lines 320-328].

Nonetheless, it has also been reported that many employed people tend to have unhealthy lifestyles because they are too busy with work to pay attention to their own health habits [41,82,83]. Moreover, work-related stress might influence the engagement in health risk behaviors, such as alcohol consumption [84,85]. The unique cultural background of Japan may also lead employed individuals to very frequently go out for drinks to interact socially and professionally with bosses and colleagues after work [86,87]; hence, those employed in Japan may be more likely to adopt unhealthy behaviors, highlighting the potential need for comprehensively analyzing the work lives of Japanese workers in order to improve their healthy behavior patterns.

31. As mentioned in some of my earlier comments, I think you need to go further when discussing the limitations of the study including the single item measure of happiness, the missing data, and the issues around using secondary sources of data.

I would like to demonstrate my appreciation for this commentary. To ensure that I followed your guidance, I have revised the Limitation section and the final portion of the Discussion section to ensure that this limitation is more clearly described, as follows [Lines 361-376] and [Lines 344-351].

**Limitations:**

Second, the valid response rate was relatively low in this study; since I used data only from a single wave (i.e., 2017) of a long-term panel dataset, it is a reality that the youngest participants at baseline are getting older. Moreover, a certain percentage of participants dropped out, and I cannot rule out the possibility of sample attrition bias. Therefore, caution should be exerted when interpreting the study findings.

Third, this study was based on a secondary data analysis; namely, I could not escape the constraints imposed by the chosen study design, especially regarding the measurement tools used by the questionnaire survey. Some of the problems that they have include unclear timeframes for measuring alcohol consumption, having items about physical activity that did not comprehensively inquire about exercise amount and intensity, or about vegetables and fruit intake that did not comprehensively inquire about the specific amounts that participants ate. Moreover, the original surveys from which my data were extracted assessed the happiness construct through a subjective, single-item scale that relied solely on self-reported responses; thus, this variable was not comprehensively explored, making it necessary for future research to use a multi-dimensional objective instrument that yields data which may allow for the development of more effective and successful strategies.

**Discussion:**

As the current study was cross-sectional, the causal relationship between health behaviors and happiness could not be examined. However, the current study confirmed the relationship between high levels of happiness and healthy behaviors and showed that health risk behaviors were more prevalent among those who had jobs or had lower SES. If healthy behaviors lead to higher levels of well-being as indicated in previous research [23, 37], it may be important for stakeholders to endeavor to assist the structuring of the private and work lives of employed Japanese individuals in a way that enables employees to adopt these healthy behaviors, a goal for which policy interventions may be required.
