# Supplementary material for: Clustering of health behaviors among Japanese adults and their association with socio-demographics and happiness
Source: PLoS One. 2022 Apr 14;17(4):e0266009. doi: 10.1371/journal.pone.0266009 (PMC9009612; doi:10.1371/journal.pone.0266009)
Supplement: Attachment — Submitted filename: Response to reviewers.docx [file pone.0266009.s002.docx]

Reviewer #1:

#1 I initially read the category of “non-employed” according to the US definition of unemployed. However, as it has been shown (Wilson & Walker, 1993, Voßemer, et al 2018) “unemployment has an adverse effect on health. This effect is still demonstrable when social class, poverty, age and pre-existing morbidity are adjusted for” Wilson & Walker 1993, p. 153). Which made me realize you non-employed may meaning something different. Can you please clarify.

Thank you for the comment. The “non-employed” category does mean “not unemployed or jobless,” but merely without occupation. Therefore, I added the annotation as follows:

non-employed; and without occupation, not jobless (p. 7, lines 172-173)

#2 Relabel Tables 3 and 4 to make clear the data define cluster 1 and cluster 2.

Thank you for the comment. I revised the labels for Clusters 1 and 2 in Tables 3 and 4 as follows:

Table 3: Association between health behavior cluster of “low-risk smoking and drinking, high vegetable intake, and regular breakfast” and socio-demographics

Table 4: Cluster 1: low-risk smoking and drinking, high vegetable intake, and regular breakfast; Cluster 2: high alcohol, poor nutrition, and exercise

#3 For some reason, I interpreted your title as focusing on positive health behaviors. But the labels of both your clusters emphasis negative practices which, although accurate, I initially interpreted as an error. My suggestion will be to change the label to emphasize the positive health practices in Cluster 1 (that you clearly list). “low risk, poor fruit and exercise with participants in it being characterized by the lowest probability for smoking, moderately low alcohol consumption, the highest vegetable intake, regular breakfast intake, as well as low fruit intake and physical activity.”

I appreciate the advice. Based on this insightful suggestion, I revised the description for Cluster 1 as follows:

“low-risk smoking and drinking, high vegetable intake, and regular breakfast,” (p. 11, line 224)

Reviewer #2: Thank you for your consideration of my comments in this revised manuscript. While I think that the edits based on my own and Reviewer 1’s comments have improved the paper, there are still a few areas where I think additional edits would further strengthen this.
In general, I think that the introduction is still slightly lacking with the justification of why this an important area to focus on. You state that it’s likely multiple health behaviours are interrelated, and that behaviour is related to happiness, but I think the introduction is missing a clear link between these two points. Why is it particularly important to look at clustered health behaviours specifically and how these are related to happiness?
I also think that the discussion could go further in depth when discussing the study findings as a whole. You state in your abstract that: “Identifying the clusters of multiple health behaviors and their effect on happiness can greatly contribute to developing strategies for enhancing happiness and improving health behaviors.” – I think that the discussion could go further when explaining and discussing this point.

Thank you for the comment. I revised the Introduction accordingly and added a brief implication for practice in the conclusion;

Scholars have found that healthy lifestyles, such as being physically active [16, 17], consuming vegetables and fruits [18-20], not smoking [21,22], and consuming alcohol in moderation [23-25], are significantly associated with happiness [26]. Thus, further engagement in health behaviors could enhance happiness [27]. Nonetheless, lifestyles are composed of multiple health behaviors. Thus, many studies have investigated the relationship between health behaviors and physical or psychological health status in isolation.

A few studies in Europe and the United States have found that multiple health behaviors tend to occur interrelatedly and that the degree of health consciousness exerts a cascading effect on all types of health behaviors [28-30]. In other words, one health behavior can trigger a chain reaction of other health behaviors, whereas the opposite is also true. Moreover, the simultaneous occurrence of multiple health behaviors has been attributed to various mechanisms, such as the commonality of multiple factors associated with such behaviors, and the fact that one behavior may be a coping strategy for another [31]. Multiple health behaviors exert synergistic effects. Therefore, research on the clustering of multiple health behaviors and their relationship with health outcomes is increasing [28,30,32-36].

Furthermore, scholars have analyzed the synergistic effects of multiple health behaviors on happiness [23, 37]. Specifically, the research on the association between multiple health behaviors and happiness has focused on younger populations of adolescents and college students [33-35]. However, less is known about multiple health behaviors across the full range of adult age population. Specifically, studies on this topic are lacking in Japan.

Intervention that targets only one health behavior can lead to consequences for improving other co-occurring health behaviors, which enhances happiness accordingly. Against this background, altering health behavior patterns could be an effective means for increasing happiness. Moreover, identifying the existing clusters of health behavior patterns and the characteristics of people belonging to these clusters would be beneficial to the formulation of intervention strategies for changing health behavior patterns. (pp. 3-4, l.59-1.84)

Based on the results, the synergistic effect of improving health behaviors can be efficiently induced by constructing measures with a compound and chain effect, instead of strategies that improve each health behavior. (p.19, l.396-398)

1. The labelling of cluster 1 is still not quite right and doesn’t particularly make sense currently. In particular ‘poor fruit’ needs changing to something more descriptive, I would suggest ‘low risk: low fruit intake, low exercise’.

Thank you for the advice. I added this following description for Cluster 1:

“low-risk smoking and drinking, high vegetable intake, and regular breakfast,” (p. 11, line 224)

2. The first line of the introduction could be restructured to improve clarity of the point you are trying to make.

Thank you for the comment. I revised the first line of the Introduction for a clearer meaning.

Happiness, which is a key concept of well-being, productivity, and quality of life, has received much scholarly attention worldwide. (p.3, l.46-l.47)

3. On a number of occasions throughout the manuscript, you use ‘I’ rather than sticking to third person (e.g. ‘this study investigated…) – these need editing into third person.

I appreciate your pointed out. I corrected first person to third person throughout the manuscript.

4. Page 6, line 111 – refers to ‘more recent ones’ – what does the ‘ones’ here refer to? Studies or behaviours? This needs clarifying.

Thank you for pointing out this aspect. I modified the first-person voice to that of the third person throughout the manuscript. “Ones” refers to studies. I have revised it from “more recent ones” to “recent studies” (p. 5, line 109)

5. The translated version of the alcohol item on p6, line 119 doesn’t make sense – I think this should read ‘how often do you consume alcohol’ or something similar.

Thank you for the comment. I revised the question about alcohol intake as follows:

“How frequently do you consume alcohol?” (p. 5, lines 121-122)

6. Line 113 refers to ‘enunciation’ when I think you mean ‘question.’

Thank you for pointing out this aspect. I corrected the word to “question” (p. 6, line 128)

7. Where does the 2 times daily fruit and vegetable cut off come from? In the UK, the standard healthy level of fruit and veg is 5 pieces of either fruit or veg per day so it would be useful to understand why this cut off was used.

Thank you for the comment. I added a note about the cut-off value for fruit and vegetable intake and the reference.

Consumption was measured with reference to the recommendation by the Ministry of Health, Labor and Welfare to eat at least two meals per day with side dishes including vegetables and fruits or an intake of at least 2SV per day of fruits and vegetables [41, 42]. (p. 6, lines 136-139)

8. Page 8, line 172, would it be possible to provide the USD equivalent to yen for the participant income levels? This would improve clarity for non-Japanese readers.

Thank you for the comment. As the question used only JPY, I could not provide the categories representing USD. Therefore, I provided the exchange rate between USD and JPY at the time of the survey.

SES (i.e., expressed in increments of 10,000 JPY; 1 USD was approximately 113 JPY at the time of the survey), (p. 7, lines 169-170)

9. You mention that missing data was excluded so there was only a 31% response rate for participants, but I think you need to provide more information about the missing data – how much data was missing for each item/participant? Was there any consideration given to imputing this data rather than excluding participants with any data missing? While you state in your response to reviewer 1 that this is acknowledged in the limitations, I could not see this in the discussion and think that it needs to be clearly stated as a limitation of the study.

Thank you for the comment. I imputed the missing data for happiness and depression with mean. However, health behaviors (categorical variables) and socio-demographic attributes (employment status and academic background) could not be imputed. Thus, responses with missing data on health behaviors, employment status, and academic background were excluded. The characteristics of the excluded participants were added to the Results section. Moreover, I cited the possibility of sample bias in the Limitations section as follows:

Participants with missing data on the socio-demographic attributes (employment status and academic background) and health behaviors were excluded. The average age of the participants with missing data was 53.98 ± 11.23 years contrary to 49.84 ± 9.16 years of the analyzed sample (*t* = −11.60, *p* < 0.000), whereas 59.2% contrary to 59.5% were male in the analyzed sample (*χ*^2^ = 114.18, *p* < 0.000). (p. 9, lines 201-205)

Moreover, differences in age and sex distribution were observed between the included participants and sample with missing data. (p. 18, lines 370-371)

10. P10 refers to regular and non-regular employment, what does this mean and what are the definitions for these two types of employment? (e.g., does ‘regular’ employment include part time employment?)

Thank you for the comment. I added the annotation about employment status as follows:

(i.e., regular; full-time with a contract for regular employment; non-regular; part-time or temporary contract; self-employed; or non-employed; without an occupation, not jobless). (p. 7, lines 171-173)

11. The two categories of breakfast eating seem to overlap each other – ‘almost every day’ to me means that participants sometimes don’t eat breakfast which would mean they would also be in the ‘not every day’ category – this labelling needs clarifying.

Thank you for the comment. I grouped the responses of breakfast-eating habit into “almost every day” and “skip 2−3 times/week.” Subsequently, I revised the description for the response category about breakfast-eating habit as follows:

Responses were coded 0 for “less than 4 days/week” and 1 for “more than 4 days/week.” (p. 6, lines 142-143)

12. Page 13-14 – the cluster 1 OR presented in the text which refers to Table 4 does not present the same OR as the figure then presented in Table 4.

Thank you for this comment. I corrected the OR and CI for Cluster 1 as follows:

OR = 1.266, 95% CI = 1.010−1.587, *p* = 0.041 (p. 12, line 246)

13. By presenting the depression findings at the very beginning of the discussion, this seems to highlight these findings as the most important results from the study. I think this paragraph needs to be moved further down the discussion and instead the key findings from the study should be highlighted in the first paragraph of the discussion.

I appreciate your pointed out. Based on your suggestion, I moved the depression findings to the end of the discussion.

Meanwhile, this study reported that 33% of the participants had depressive tendencies. In a recent study, approximately 25% of Japanese adults suffer from mental health problems, such as anxiety disorders and mood disorders [94]. Therefore, although the prevalence of depression in the samples of the present study was slightly higher than that for mental health problems in the Japanese population, it did not deviate significantly from the trend. (p. 17, lines 357-361)

14. The first mention of cluster 1 and 2 in the discussion needs to remind the reader of what both of these clusters represent – I know that you have later defined these in the discussion, but I think it’s important to do this as soon as these are mentioned in the discussion section.

Thank you for this comment. Based on the suggestion, I added the definitions for Clusters 1 and 2 in the first part of the Discussion section.

Based on the distribution of health behavior, the participants in Cluster 1 (low-risk smoking and drinking, high vegetable intake, and regular breakfast) exhibited low health risk behaviors than those in Cluster 2 (high alcohol, poor nutrition, and exercise) (p. 19, lines 396-398)

15. Page 16, You refer to the fact that younger participants having an unhealthier lifestyle as a health awareness issue – I think it also needs to be acknowledged here that this is likely to be a time constraint/societal issue rather than just being related to awareness.

Thank you for the comment. I added a discussion on the tendency of the younger participants toward an unhealthy lifestyle as follows:

Nevertheless, other surveys have found that people aged in their 30s and 40s tend to be more constrained in terms of time and are less aware of their health due to increased roles and responsibilities in family and work life [42,62], (p. 15, lines 287-289)
