# Supplementary material for: Clustering of health behaviors among Japanese adults and their association with socio-demographics and happiness
Source: PLoS One. 2022 Apr 14;17(4):e0266009. doi: 10.1371/journal.pone.0266009 (PMC9009612; doi:10.1371/journal.pone.0266009)
Supplement: Attachment — Submitted filename: Response to Reviewers.docx [file pone.0266009.s003.docx]

Review Comments to the Author
Please use the space provided to explain your answers to the questions above. You may also include additional comments for the author, including concerns about dual publication, research ethics, or publication ethics. (Please upload your review as an attachment if it exceeds 20,000 characters)

Reviewer #1: While the author responded to the reviewers’ comments and provide clarification, significant issues remain.

#1

- Line 131 Make it clear if the classification is for current smokers vs non-smokers or never smokers vs ever smoker.

**Response**: Thank you for your suggestion. I have included additional information about grouping of smoking status as follows:

Responses were coded as 1 for current non-smokers (responded “I have quit smoking” or “Never”) and 0 for current smokers (responded “I smoke” or “I smoke sometimes”). (p. 7, lines 132–133)

#2

- Line 138 spell out 2SV

**Response**: Thank you for your comment. I have spelled 2SV out to two servings (p. 6, line 140)

#3

- Line 164 “Cut-off scores of 3 and 3 were used for depression.” Clarify

**Response**: Thank you for your comment. Based on your comment, I have added information about cut-off point of the GHQ

The cut-off point of the GHQ was set at 2/3 [46,47]; those scoring 3 points or higher were considered to indicate depression. (p. 8, lines 165–167)

#4

- Line 171-173 your clarification regarding employment status does not solve the problem. What does without an occupation mean? meaning out of the labor force? Provide a clear definition, I found these two articles very informative and wondered if that is what you were talking about.

Gnambs, T., Stiglbauer, B., & Selenko, E. (2015). Psychological effects of (non) employment: A cross‐national comparison of the United States and Japan. Scandinavian Journal of Psychology, 56(6), 659-669.

Genda, Y. (2007). Jobless youths and the NEET problem in Japan. Social Science Japan Journal, 10(1), 23-40.

**Response**: Thank you for your comment and for suggesting relevant literature. I have revised “without an occupation” to “non-employed” based on the recommended literature.

(… non-employed: those who are neither workers nor unemployed, such as full-time housewives, students or retirees) [48, 49]. (pp. 7-8, lines 175–176)

#5

- Line 222 - What about labeling your clusters something brief but informative and consistent, rather than Cluster 1 and Cluster 2. Your inconsistent use of descriptors to show directionality for both healthy (low fruit intake) and unhealthy (low risk smoking) behaviors makes it harder to keep the direction of the findings in order.

Even your conclusion is confusing: Thus, belonging to the cluster of low-risk health behaviors (low fruit intake, low exercise) may be significantly linked to high levels of happiness. What is the role of “low-risk smoking and drinking, high vegetable intake, and regular breakfast”?

**Response**: Thank you for your suggestion. I agree with you and have attempted to revise the labels of Clusters 1 and 2 to reflect their characteristics to the best of my ability. I have also revised the discussion of the characteristics of both clusters. Accordingly, I have used the new labels instead of Cluster 1 and Cluster 2 after the result section.

Cluster 1 (n = 817; 52.3%) was characterized by the lowest probability for smoking, moderately low alcohol consumption, highest vegetable intake, and regular breakfast intake and was described as “low substance use and good dietary habit.” Cluster 2 (n = 737; 47.7%) was described as “high alcohol, poor nutrition, and inactive,” with participants characterized by high levels of alcohol consumption, low vegetable intake, very low fruit intake, and very low physical activity. (p. 12, lines 225–231)

Based on the distribution of health behaviors, participants in the “low substance use and good dietary habit” cluster exhibited low health risk behaviors than those in the “low substance use and good dietary habit” cluster. Having breakfast regularly may be associated with high vegetable consumption [51-53]. Previous studies have suggested that those who show high health awareness are likely to have some healthy behaviors simultaneously [37,54–57]. Thus, the “low substance use and good dietary habit” cluster, comprising individuals who are non-smokers, moderate drinkers of alcohol, eat vegetables nearly every day, eat breakfast nearly every day, even though with low fruit intake, and are physically inactive, was considered a health behavior pattern. (p. 15, lines 258–267)

#6

- Line 230 should be lower

**Response**: Thank you for your pointing this out. I replaced “low” with “lower.” (p. 12, line 231)

#7

- Line 236 “regular employees (OR = 0.540, 95% CI = 0.341-0.857, p = 0.009), non-regular employees (OR = 0.576, 95% CI = 0.368-0.903, p = 0.016), and self-employed individuals (OR = 0.539, 95% CI = 0.322-0.901, p = 0.018) displayed lower odds of belonging to Cluster 1.” Does it mean that working people engage in poor health practices? Say it clearly in the discussion, there is a subtle reference only.

**Response**: Thank you for your comment. As you pointed out, this study found that working people could have poor health practices. Therefore, I have revised the discussion on the association between clusters of health behaviors and employment status.

Additionally, other surveys have found that people in their 30s–40s tend to face time constraints and are less aware of their health due to increased roles and responsibilities in family and work life [42,71]. That could explain the lack of association between younger age group and the “low substance use and good dietary habit” cluster and it might also indicate that employed people were more likely to be in the “high alcohol, poor nutrition, and inactive” cluster. The present study demonstrated that the employed Japanese population was more prone to unhealthy behaviors. A large proportion of the insured, working adults failed to meet the recommendations for health behaviors in the US [72]. Other studies have also reported that many employed people tend to observe unhealthy lifestyles, because they are extremely busy with work and fail to pay attention to their health habits [42,73,74]. Moreover, work-related stress may influence engagement in health risk behaviors, such as alcohol consumption [75,76]. The work culture may also lead employed individuals to frequently go out for drinks to interact socially and professionally with bosses and colleagues after work [77,78]. Hence, employed individuals in Japan may be more likely to adopt unhealthy behaviors, which highlights the potential need for a comprehensive analysis of the work lives of Japanese workers to improve health behavior patterns. (p.17, lines 307–321)

#8

- Line 255 “However, those in Cluster 1 were not entirely healthy” Actually you do not know how healthy they are, just know about their healthy practices. So maybe something like: “However, even individuals engage in "health living" (cluster 1), did not embrace all health recommendations equally.”

**Response**: Thank you for your pointing it out. Based on your advice, I have revised as follows:

However, individuals in this cluster that engaged in “health living” did not embrace all health recommendations equally. (p. 15, lines 266–267)

#9

- Line 261 Replace “findings” for “pattern”

**Response**: Thank you for your pointing it out. I replaced “findings” with “pattern.” (p. 15, line 269)

#10

- Line 272 “The participants in Cluster 2 also exhibited moderate smoking” What does moderate smoking mean?!! Smoking is smoking!

**Response**: Thank you for your insightful comment. I revised here as follows:

“High alcohol, poor nutrition, and inactive” cluster also exhibited higher current smoking habit and lower vegetable intake than those in the “low substance use and good dietary habit” cluster. (p. 16, lines 283–285)

#11

- Lines 285 – 286 Reword older ones vs younger ones, poorly worded

**Response**: Thank you for your insightful comment. I reworded these words to be as appropriate as possible.

Japanese people in their 50s–60s tend to display increased levels of health awareness and adopt healthier lifestyles compared with the people in their 30s–40s [42,66]. (p. 16, lines 298–299)

#12

- Line 287 – 289 State it clearly. Is this the reason why working people are more likely to be in cluster 2?

**Response**: Thank you for your comment. Related to comment #7, this study found that working individuals could follow poor health practices, and I speculated the reasons for this as follows:

Additionally, other surveys have found that people in their 30s–40s tend to be face time constraints and are less aware of their health due to increased roles and responsibilities in family and work life [42,71]. That could explain the lack of association between younger age group and “low substance use and good dietary habit” cluster, and it might also indicate that employed people were more likely to be in “high alcohol, poor nutrition, and inactive” cluster. The present study demonstrated that the employed Japanese population was more prone to unhealthy behaviors. A large proportion of the working insured adults failed to meet the recommendations for health behaviors in the US [72]. Other studies have also reported that many employed people tend to observe unhealthy lifestyles, because they are extremely busy with work and fail to pay attention to their health habits [42,73,74]. Moreover, work-related stress may influence engagement in health risk behaviors, such as alcohol consumption [75,76]. The work culture may also lead employed individuals to frequently go out for drinks to interact socially and professionally with bosses and colleagues after work [77,78]. Hence, employed individuals in Japan may be more likely to adopt unhealthy behaviors, which highlights the potential need for a comprehensive analysis of the work lives of Japanese workers to improve health behavior patterns. (p.17, lines 307–321)

#13

- Line 306 – 309 – One can also speculate, higher education, higher income, more resources, more free time …

**Response**: Thank you for your insightful comment. I agree with your comment that those with higher level of education or higher income could have more resources for health promotion. However, it was unclear whether such people could have free time to practice healthy habits. Therefore, I have revised the text from the standpoint of education level and SES as follows:

According to previous studies, high levels of education may be significantly associated with health behaviors [30,83], which is similar to the results obtained by the current study. In Japan, the level of education is related to health behaviors, where high levels of education are associated with more physical exercise routines [84], lower problem drinking [85], and lower obesity [86]. In addition, the current study confirmed the association between multiple healthy behaviors and the higher SES. This result was also congruent with literature [80,87]. Scholars have speculated that this finding is due to the fact that the higher the level of education or the higher income, the more knowledge or resources people utilize for health [88,89]. Therefore, they are more likely they are to engage in health-promotion activities and also gain access to useful resources that can improve health. This indicates the need to examine the content of education in schools to ensure that young people, regardless of their SES, gain appropriate knowledge about health and health behaviors from the stage of primary education. (pp. 17–18, lines 327–338)

#14

- Line 329 “many studies” but cite only 1 – reference 87

**Response**: Thank you for your pointing out. I apologize for the confusion. I have revised accordingly, as follows.

One systematic review and metanalysis on health behavior patterns reported no sex differences in health risk behaviors [90] (p. 18, lines 339–340)

#15

- Line 357 replace “reported” with found

**Response**: Thank you for pointing this out. I replaced “reported” with “found.” (p. 19, line 367)

Reviewer #2

- Thank you for addressing my comments in this revision. I have one further (very minor) suggestion which is that on P3, line 46 I would replace the word ‘concept’ with ‘facet’.

**Response**: Thank you for your advice. I have replaced “concept” with “facet.” (p. 4, line 47)
